# Numerical subgrid Bi-cubic methods of partial differential equations in image segmentation

**DOI:** 10.1038/s41598-024-54855-7

**Published:** 2024-04-10

**Authors:** Dongyung Kim

**Affiliations:** 1 Department of Mathematics, Phoenix College, Phoenix, AZ 85013 USA; 2 Department of Mathematical Sciences, Seoul National University, Seoul, Republic of Korea

**Keywords:** Partial differential equations, Image segmentation, Numerical methods, Medical research, Engineering, Mathematics and computing, Optics and photonics, Medical research, Engineering, Mathematics and computing, Optics and photonics

## Abstract

Image segmentation is a core research in the image processing and computer vision. In this paper, we suggest a Bi-cubic spline phase transition potential and elaborate a Bi-Cubic spline phase transition potential development. In the image segmentation, we develop the new approach to apply the novel computational fluid dynamics in the boundary with subgrid. The numerical subgrid Bi-cubic method with Bi-Cubic spline for minimizing the piecewise constant energy functional is very efficient, robust and fast in the image segmentation with a multispecies multiphase segmentation models. The subgrid Bi-cubic spline is applied on the boundary with subgrid and the regular grid is applied on the non-boundary. The model generates a multispecies multiphase distribution with Bi-Cubic spline and we can extract the image segments with multispecies multiphase. Finally, we analyze the models and show the numerical results. Numerical results are presented with OCR (Optical Character Recognition) and the medical image.

## Introduction

Computational image segmentation role is fundamental in the detection, the matching and the tracking from image processing and robot vision^1^. It is the preprocessing procedure for detecting the object^2,3^. Numerical Techniques in MATLAB is helpful to simulate the image segmentation code^4^. The image processing is based on the computational fluid dynamics skills^5^. In the computational fluid dynamics, there are two major solver to solve the partial differential equations. One is the finite difference scheme (FDS) and the other is the finite element scheme (FES). FES is from Galerkin Scheme. In FES, there are many type of spline to generate Soblev space. We develop a spline to apply from the computational fluid dynamics to image segmentation. Because image intensity is flowing from the high density to the lower density with diffusion. The aim of the image segmentation is to extract the distinct objects and to recognize the object. Our motivation and innovation is to obtain the satisfactory results on the boundary grids. So we suggest and develope the new Bi-cubic spline methods. It is different on the boundary grids from the traditional methods. This is key role with our Bi-cubic spline of image intensity interface on the domain. Image segmentation is also an important role in face recognition. In the face recognition, the optimized classification method can be applied for our futre work^6,7^. The application of the image segmentation is diverse. In the OCR image, the segmentation is key role to recognize it from the media or CCTV^8^. In the medical image, it is critical to detect and decompose the tumor part image through the full image. In the security or defense application, it contribute to match the image from the streaming movie^9^. Their references are in Refs.^3,10–15^.

Since the image has multispecies multiphase property, multispecies multiphase models with Bi-cubic spline are suggested. We describe the proposed numerical subgrid Bi-cubic spline method between computational fluid dynamics^16–18^ and image intensity computation. In detail, we suggest a Bi-cubic spline phase transition potential and show the numerical results in the several application for image segmentation. Previously, in the image inpainting, the computational fluid dynamics scheme is applied to obtain the conservation law in image intensity. In the image, the critical problem is from the boundary or boundary grid. Our suggested Bi-cubic spline approach in the boundary of the image segmentation is working well. We obtain the satifactory numerical results in image segmentation.

The organization of the paper is as follows. Sect. “Introduction” Bi-cubic spline phase transition potential and methods are described in “Methods”. The numerical results of the image segmentation are presented in “Results”. Conclusion and discussion are given in “Conclusions and discussion”.

## Methods

The multiphase approximation model is proposed with *K*-phase fields from minimizing the Mumford-Shah functional and Allen-Chan equation is applied for the length of the curve *C*^1,19^. Additionally, we proposed the new model with a Bi-cubic spline potential. This suggested model has a different Bi-cubic spline phase transition potential $BiC(\langle \phi \rangle )$ instead of the phase transition potential from the original model. The model of energy functional is the following:

1 $$\begin{aligned} {{{E}}({\phi })}= \int _\Omega \left( \frac{ BiC(\langle \phi \rangle )}{{{\varepsilon ^2}}} + \frac{{|\nabla \phi {|^2}}}{2}+G_{k}(\phi ,I_{0})\right) d{\textbf{x}}, \end{aligned}$$

$\phi$ is phase-field which is meaning the mixing rate (Atwood number) in computational fluid dynamics^16,17,18^. $\langle \phi \rangle$ is defined by $\phi -[\phi ]$, $[\phi ]$: the largest integer not greater than $\phi$. In the phase transition, we applied a Bi-cubic spline in the image domain and image intensity interface. We suggest and define a Bi-cubic spline phase transition potential $BiC(\langle \phi \rangle )$ as the following:

2 $$\begin{aligned} { \displaystyle BiC(\langle \phi \rangle )} = {\left\{ \begin{array}{ll} \frac{(2+\langle \phi \rangle ^3)}{150} &{} if \nonumber -1 \le \langle \phi \rangle \le \frac{-1}{2}, \\ \frac{(4-6\langle \phi \rangle ^2-3\langle \phi \rangle ^3)}{150} &{} if \ \frac{-1}{2}< \langle \phi \rangle \le 0, \\ \frac{(4-6\langle \phi \rangle ^2+3\langle \phi \rangle ^3)}{150} &{} if \ 0< \langle \phi \rangle \le \frac{1}{2}, \\ \frac{(2-\langle \phi \rangle ^3)}{150} &{} if \ \frac{1}{2} < \langle \phi \rangle \le 1, \\ 0 &{} otherwise. \end{array}\right. } \\ \end{aligned}$$

From the energy funtional, the first term denomerator’s $\varepsilon$ is the gradient energy coefficient to the interfacial energy force. In the image application, we denote *I* for image intensity. $I_{0}$ is the normalized initial image intensity. $\Omega$ is the image whole domain of all collections of pixels. From the modified model, the second term, $\frac{{|\nabla \phi {|^2}}}{2}$ is variation of the transition with the spline (2) between smoothing levels and high oscillation levels. The third term is the source term to fit the model^1^.

3 $$\begin{aligned} G_{k}(\phi ,I_{0})=\frac{\lambda }{2}\sum _{k=0}^{K-1} {\left| C_{k}-I_{0} \right| }^2\text{ sinc}^2(\phi -k). \end{aligned}$$

^1^

**Figure 1:**
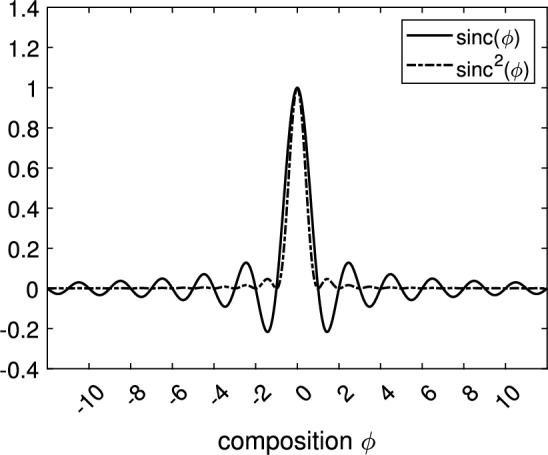
$$\text{ sinc }(x)={\sin (\pi \phi )}/{(\pi \phi )}$$ and $$\text{ sinc}^2(\phi )=\sin ^2(\pi \phi )/(\pi \phi )^2$$.

$\text{ sinc }(\phi )={\sin (\pi \phi )}/{(\pi \phi )}, \phi \in \mathbb {R}.$ Refer to Fig.1. $\lambda$ is a nonnegative parameter. $I_{0}$ is the initial image after normalization. Also $C_{k}$ is the averages of $I_{0}$ in the regions $({\phi \in [\frac{k}{K},\frac{k+1}{K}]})$ ($k=0,1,...,K-1$) and defined as the following

4 $$\begin{aligned} {C_{k}} =\frac{\int _\Omega {{I_{0}(\mathbf{{x}})}\text{ sinc}^2(\phi (\mathbf{{x}})-k)d\mathbf{{x}}} }{\int _\Omega {\text{ sinc}^2(\phi (\mathbf{{x}})-k)d\mathbf{{x}}}}. \end{aligned}$$

^1^

When $\phi$ flow attain to a steady state in the image intensity flow, the $k+1$ level set will become the contour for each individual $k_{th}$ phase. For this purpose we get the following gradient descent flow equation:

5 $$\begin{aligned} {\phi _t} = -\frac{ BiC'(\langle \phi \rangle )}{{{{\varepsilon ^2}}}} + \Delta \phi - \lambda \sum _{k=0}^{K-1}|C_{k}-I_{0}|^2 \left( \frac{\sin (2\pi ( \phi - k ))}{\pi ^2{ (\phi - k )^2}} -\frac{2\sin ^2({\pi ( \phi - k )})}{\pi ^3{( \phi - k )^3}}\right) \end{aligned}$$

We show numerical subgrid Bi-cubic spline method for the Mumford-Shah functional. It is the modified model methods with the image intensity in 3*D* spline $=$ 2*D* spline X 2*D* spline.

The image intensity function is in three dimensional space. The image intensity function values is from 0 to 255 but it is rescaled from 0 to 1. First, we discretize two dimensional space of the domain, $\Omega = (a,b) \times (c,d)$ as a evenly discretized regular domain setting. Let $N_x$ and $N_y$ be positive even integers, $h = \frac{ (b-a)}{N_x}$ be the uniform mesh size, and $\Omega _{h} = \{ (x_i,y_j) : x_i = (i-\frac{1}{2})h,~ y_j = (j-\frac{1}{2})h, ~ 1 \le i \le N_{x}, ~ 1 \le j \le N_y \}$ be the set of the cell centers for the computational domain. Through the cell centers, we generate the subgrid on the domain. Let $\phi _{ij}^n$ be approximations of $\phi ({x_i},{y_j},n \Delta t)$, where $\Delta t=T/N_t$ is the time step, *T* is the final time, and $N_t$ is the total number of time steps. By using these grid and subgrid on the boundary, we propose the following numerical subgrid Bi-cubic spline methods.

$$\begin{aligned} \frac{\phi ^{n+1}-\phi ^{n-1}}{2 \Delta t}= & {} -\frac{ BiC'(\langle \phi ^{n+1}\rangle +\langle \phi ^{n-1}\rangle )}{2 \varepsilon ^2} +\Delta _{d}\phi ^{n}\\{} & {} -\lambda \sum _{k=0}^{K-1}|C_{k}-I_{0}|^2 \left( \frac{\sin (2\pi ( \phi ^n- k ))}{\pi ^2{( \phi ^n- k )^2}} - \frac{2\sin ^2({\pi ( \phi ^n- k )})}{\pi ^3{( \phi ^n- k)^3}}\right) \end{aligned}$$

In Eq. (6), RHS’ the first term with Bi-cubic spline can be transformed to the following:

$$\begin{aligned} BiC'(\langle \phi ^{n+1}\rangle )= & {} \frac{1}{2} ( \phi ^{n+1} ((\phi ^{n+1})^2-2 \phi ^{n+1} +1)+ (\phi ^{n+1})^3 -(\phi ^{n+1})^2) \\= & {} (\phi ^{n+1})^3-\frac{3}{2} (\phi ^{n+1})^2 + \frac{1}{2} \phi ^{n+1} \end{aligned}$$

and

$$\begin{aligned} BiC'(\langle \phi ^{n-1}\rangle )= & {} \frac{1}{2} ( \phi ^{n-1} ((\phi ^{n-1})^2-2 \phi ^{n-1} +1)+ (\phi ^{n-1})^3 -(\phi ^{n-1})^2) \\= & {} (\phi ^{n-1})^3-\frac{3}{2} (\phi ^{n-1})^2 + \frac{1}{2} \phi ^{n-1}. \end{aligned}$$

Finally, the RHS of the Eq. (6) with Bi-cubic spline can be transformed to the following: $A=(\phi ^{n+1})^3 + (\phi ^{n-1})^3$,

6 $$\begin{aligned} BiC= & {} (\phi ^{n+1})^2 - (\phi ^{n-1})^2 - \left( \frac{ A -\frac{3}{2} BiC + \frac{1}{2} (\phi ^{n+1} + \phi ^{n-1} ) }{ 2 \varepsilon ^2 } \right) +\Delta _{d} \phi ^{n}\nonumber \\{} & {} -\lambda \sum _{k=0}^{K-1}|C_{k}-I_{0}|^2 \left( \frac{\sin (2\pi ( \phi ^n- k ))}{\pi ^2{( \phi ^n- k )^2}} - \frac{2\sin ^2({\pi ( \phi ^n- k )})}{\pi ^3{( \phi ^n- k)^3}}\right) \end{aligned}$$

**Figure 2:**
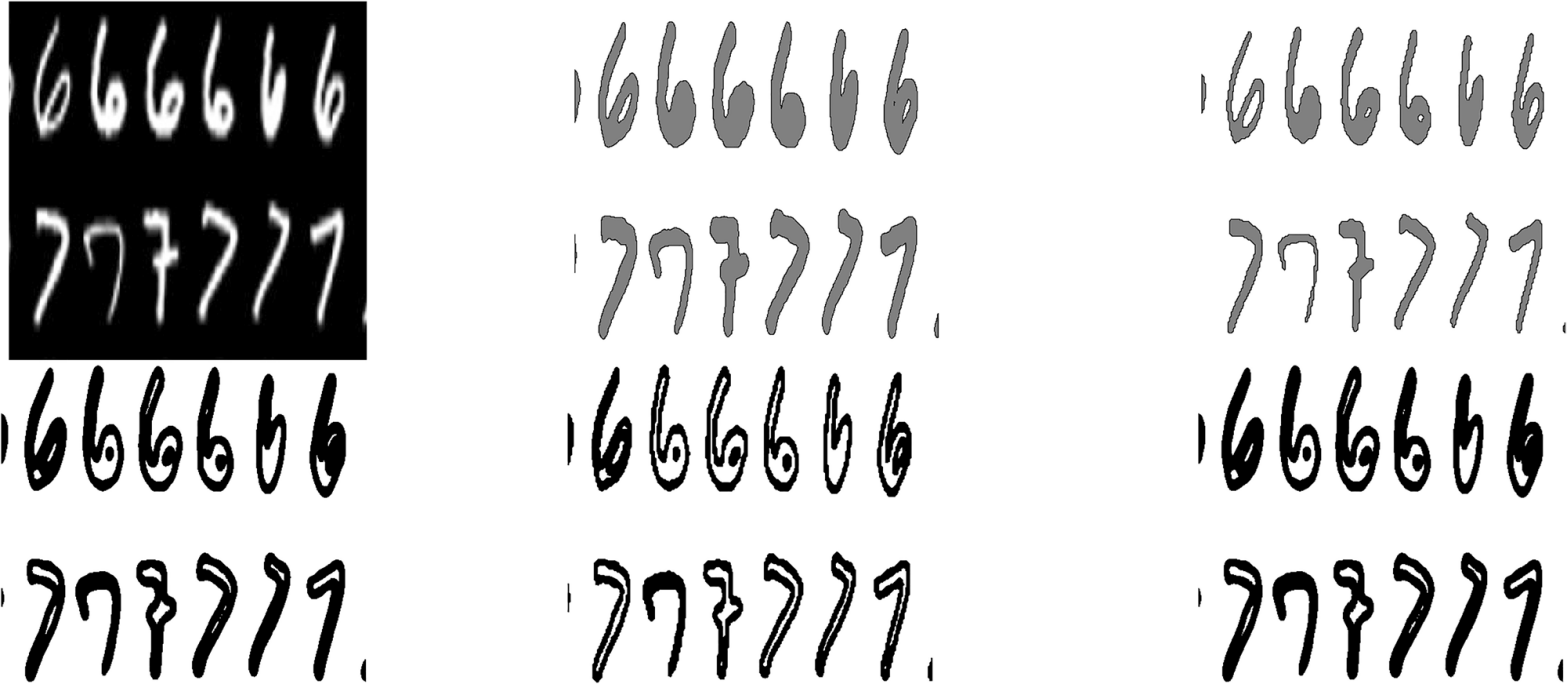
Figures from Top Left to Right : (**a**) Original image, (**b**) $\phi =0.5$, (**c**) $\phi =1.5$ with $tanh^{-1}(0.9)$. Figures from Bottom Left to Right : (**d**) $\phi =1.5$ with $tanh^{-1}(0.45)$, (**e**) $\phi =0.5$–1.5 with $tanh^{-1}(0.9)$, (**f**) $\phi =0.5$–1.5 with $tanh^{-1}(0.45)$ The images by using our proposed model to OCR (Optical Character Recognition). After ten iterations, we have the segmentation image. The computational domain $\Omega =(0,1)\times (0,1)$ with a $256\times 256$ mesh. The interface parameter $\varepsilon _{2}$ and time step $\Delta t=5$E$-6$ are used.

Briefly, in our first numerical result, we will show that above computation results in the OCR (Optical Character Recognition) test, shown in Fig. 2.

Figure 2 shows the application of the images by using our proposed model to OCR (Optical Character Recognition). After ten iterations, we have the segmentation image. The computational domain $\Omega =(0,1)\times (0,1)$ with a $256\times 256$ mesh. The interface parameter $\varepsilon _{2}$ and time step $\Delta t=5$E$-6$ are used.

We show the suggested Bi-cubic spline algorithm procedure to solve the image segmentation problems (6) which is the following:

**Step 1.**: Normalize the image intensity values [0,1] from [0, 255].
**Step 2.**: Compute ${BiC'(\langle \phi ^{n+1}\rangle +\langle \phi ^{n-1}\rangle )}$ by using Bi-cubic spline phase transition and the gradient energy coefficient to the interfacial energy force, $\varepsilon$.
**Step 3.**: Compute $\phi ^{n-1}$, $\phi ^{n+1}$,$\phi ^{n-\frac{1}{2}}$, $\phi ^{n+\frac{1}{2}}$ and update all computational domain with the subgrid boundary.
**Step 4.**: Compute (6) and update the interior grid cells on the domain and subgrid cells on the boundary domain.

## Results

In this section, we present some numerical results by our proposed modified models with Bi-cubic spline. We applied this models, the numerical scheme and solutions to the medical image. This methods can be extended and applied to the panoramic image, the blurred image and the shaken image for our future works. It can be realized that our proposed scheme is fast, accurate, and robust. In our numerical experiments, we normalize the given image intensity *I* with the spline (2) as $I_{0}=\frac{I-I_{min}}{I_{max}-I_{min}}$, where $I_{max}$ and $I_{min}$ are the maximum and the minimum values of the given image, respectively. Through this normalization with the spline (2), the initial image intensity function, $I_0 \in [0,1]$. Then the mixing interfacial regions with RGB or Gray level $\in [0,1]$. The interfacial concentration rate with the spline (2) varies from 0.1 to 0.9. The approximate distance measurement can be calculated by $4\sqrt{2} \varepsilon \tanh ^{-1}(0.9)$. We take the gradient energy parameter for the interfacial energy force, $\varepsilon _m = \frac{hm}{\alpha },$ with $\alpha ={4\sqrt{2} \tanh ^{-1}(0.9)}$ and *m* gird points for subgrid, *h* uniform grid steps. After we initialized $\phi =KI_{0}$ with the spline (2) and *K*-phase, we obtain the reasonable numerical results. See the following figures.

**Figure 3:**
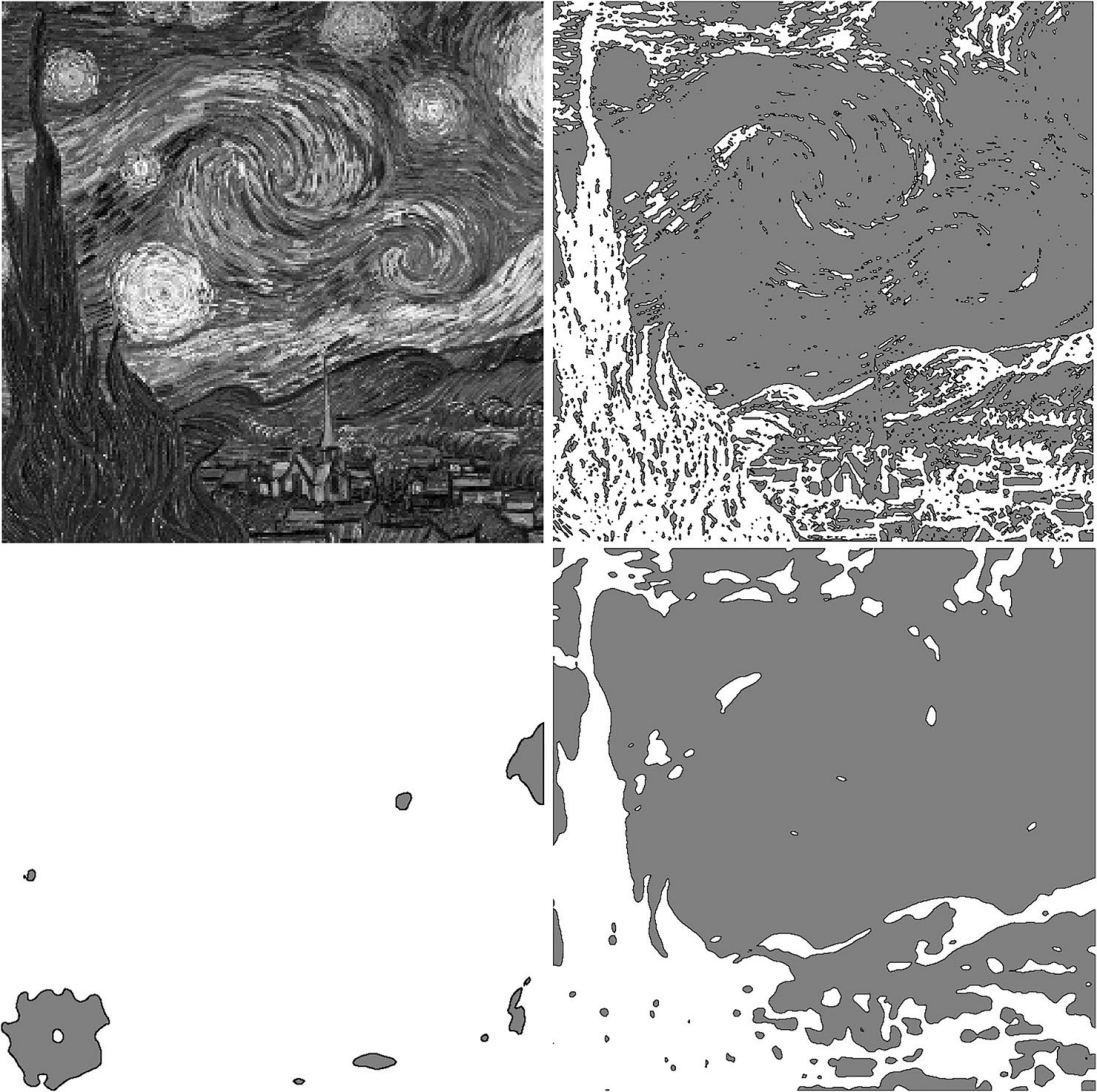
Figures from Top Left to Top Right : (**a**) Original image, (**b**) $\phi =0.5$, Figures from Bottom Left to Bottom Right: (**c**) $\phi =1.5$, (**d**) $\phi =0.5$–1.5 The original image is from the OpenCV Website^20^. The original is the open image. After ten iterations, we have the segmentation image. The computational domain $\Omega =(0,1)\times (0,1)$ with a $256\times 256$ mesh. The interface parameter $\varepsilon _{2}$ and time step $\Delta$ t = $5 \times 10^{-6}$.

Figure 3 shows the several images results with our model to the oil painting image. The original image is from the OpenCV Website^20^. The original is the open image. After ten iterations, we have the segmentation image. The computational domain $\Omega =(0,1)\times (0,1)$ with a $256\times 256$ mesh. The interface parameter $\varepsilon _{2}$ and time step $\Delta$ t = 5x$10^{-6}$.

**Figure 4:**
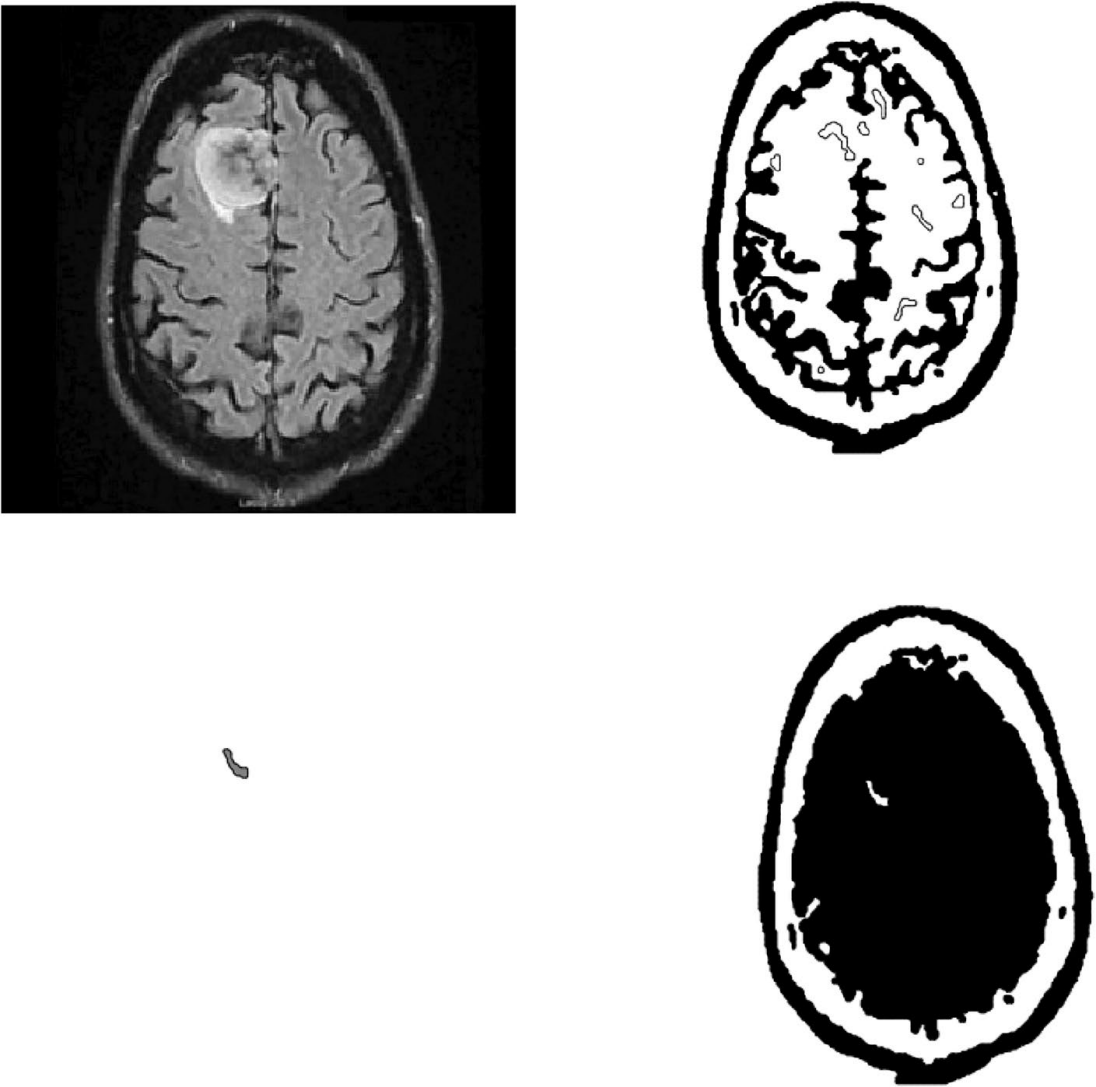
Figures from Top Left to Top Right : (**a**) Original image, (**b**) $\phi =0.5$, Figures from Bottom Left to Bottom Right (**c**) $\phi =1.5$, (**d**) $\phi =0.5$–1.5. The original image is from^21^. The original is the malignant (cancerous) image. After ten iterations, we have the segmentation image. The computational domain $\Omega =(0,1)\times (0,1)$ with a $256\times 256$ mesh. The interface parameter $\varepsilon _{2}$ and time step $\Delta$ t = $5 \times 10^{-6}$.

**Figure 5:**
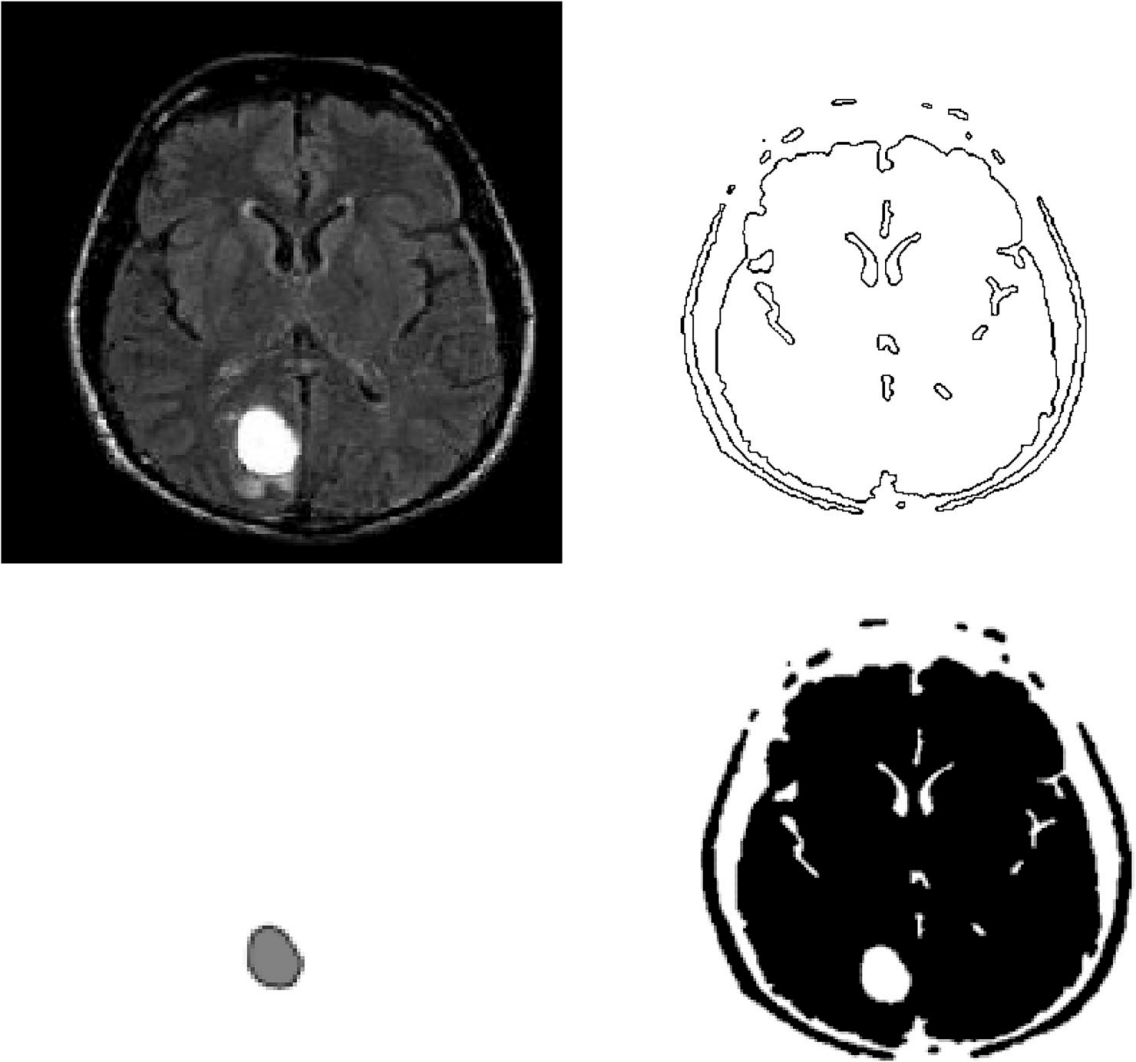
Figures from Top Left to Top Right : (**a**) Original image, (**b**) $\phi =0.5$, Figures from Bottom Left to Bottom Right (**c**) $\phi =1.5$, (**d**) $\phi =0.5$–1.5. The original is the malignant image. After ten iterations, we have the segmentation image. The computational domain $\Omega =(0,1)\times (0,1)$ with a $256\times 256$ mesh. The interface parameter $\varepsilon _{2}$ and time step $\Delta$ t = $5 \times 10^{-6}$.

Figures 4 and 5 shows the application of the images by using our proposed model to a magnetic resonance imaging (MRI) brain image.

## Conclusions and discussion

In this paper, we propose new image segmentation methods with the Bi-cubic spline (2) which is from the computational fluid dynamics scheme, the finite element scheme (FES). In the computational fluid dynamics (CFD), there are many novel approach to solve the partial differential equations. This is valuable time to apply image segmentation from the computational fluid dynamics. Our suggested methods are fast, robust and accurate. Therefore, we obtain the satisfactory results in the image segmentation. Especially, OCR (Optical Character Recognition) and the medical image are applied in the application image. From OCR images, we obtian the satisfactory results through adjusting $\phi$ values. From the brain images, we obtain the tumor part segmentation from the malignant image by adjusting $\phi$ values. We have some difficulty to obtain the real physical and medical images to investigate the data and methods comparison. This methods can be also apply to the satellite image. The several parameter of the suggested models can be adjusted to have the demanding results in the segmentation of image. This new image segmentation methods can be applied to develope the artificial intelligence about detecting several objects or obstacles. In the future works, we can develop the conservation law scheme with the volume fraction in image inpainting with the computational fluid dynamics.

## Data Availability

All data generated or analyzed during this research are included in this paper. This data is available from this paper. If you need to have further information, then email to the author.
